# Renewal of the Major Fields of New-Generation Computing

**DOI:** 10.1007/s00354-023-00206-5

**Published:** 2023-02-20

**Authors:** Sven Groppe

**Affiliations:** University of Lübeck, Lübeck, Germany

## Artificial Intelligence to Fight COVID-19 and Other Pandemics

The COVID-19 pandemic was the first pandemic with an enormously dramatic global impact after the world went digital. Never before we got so much data about a disease in such a timely fashion [3]. Humanity is still fighting the COVID-19 pandemic with this large-scale data collection and timely research based on it. The large-scale data collection meets the requirements of artificial intelligence and machine learning approaches for applications based on various analyses of the COVID-19 pandemic. It is not surprising that these types of applications have been developed in a very short time after the effects of the pandemic became clear and more and more sophisticated applications are emerging. Furthermore, while the predicted duration of the COVID-19 pandemic is only few years [2], the research based on the data of the COVID-19 pandemic may last for decades [1] and possibly even be reconsidered each time a new pandemic arises.

After organizing two special issues [4, 5] targeting submissions dealing with artificial intelligence in global epidemics, the New Generation Computing journal continuously receives a high number of submissions in this area. Hence, it has been decided to establish an own area in the journal for these submissions. Furthermore, the new area should also cover contributions about artificial intelligence for other diseases with pandemic dimensions as well. In this way, we want to offer researchers a platform for publishing results about any new pandemic and mitigate its destructive effects. We welcome contributions to the following (non-exclusive) list of topics:

- Infectious Disease Forecasting including Effects of Confinements and Vaccination
- AI
  - for Increasing Epidemic Preparedness in Public Health,
  - for the Detection of Diseases and
  - in Genome Sequencing
- Role of AI in Contact Tracing
- AI-Assisted Testing
- Generating Recommendations for Individuals’ Health
- Situation Awareness
- Sentiment Analysis and Trustworthiness of Information During Epidemics

Area Editor Sven Groppe University of Lübeck, Germany
